# Activation of efficient DNA repair mechanisms after photon and proton irradiation of human chondrosarcoma cells

**DOI:** 10.1038/s41598-021-03529-9

**Published:** 2021-12-16

**Authors:** Birgit Lohberger, Dietmar Glänzer, Nicole Eck, Sylvia Kerschbaum-Gruber, Elisabeth Mara, Simon Deycmar, Tobias Madl, Karl Kashofer, Petra Georg, Andreas Leithner, Dietmar Georg

**Affiliations:** 1 Department of Orthopedics and Trauma, Medical University of Graz, Auenbruggerplatz 5-7, 8036 Graz, Austria; 2 Department of Radiation Oncology, Medical University of Vienna, 1090 Vienna, Austria; 3 MedAustron Ion Therapy Center, 2700 Wiener Neustadt, Austria; 4 University of Applied Science, 2700 Wiener Neustadt, Austria; 5 Laboratory for Applied Radiobiology, University Zurich, 8006 Zurich, Switzerland; 6 Gottfried Schatz Research Center for Cell Signaling, Metabolism and Aging Molecular Biology and Biochemistry, Medical University of Graz, 8010 Graz, Austria; 7 BioTechMed-Graz, 8010 Graz, Austria; 8 Institute of Pathology, Medical University of Graz, 8010 Graz, Austria

**Keywords:** Bone cancer, Cancer metabolism, Sarcoma, Cancer, Cell biology, Cell death, Molecular biology, DNA damage and repair

## Abstract

Although particle therapy with protons has proven to be beneficial in the treatment of chondrosarcoma compared to photon-based (X-ray) radiation therapy, the cellular and molecular mechanisms have not yet been sufficiently investigated. Cell viability and colony forming ability were analyzed after X-ray and proton irradiation (IR). Cell cycle was analyzed using flow cytometry and corresponding regulator genes and key players of the DNA repair mechanisms were measured using next generation sequencing, protein expression and immunofluorescence staining. Changes in metabolic phenotypes were determined with nuclear magnetic resonance spectroscopy. Both X-ray and proton IR resulted in reduced cell survival and a G2/M phase arrest of the cell cycle. Especially 1 h after IR, a significant dose-dependent increase of phosphorylated γH2AX foci was observed. This was accompanied with a reprogramming in cellular metabolism. Interestingly, within 24 h the majority of clearly visible DNA damages were repaired and the metabolic phenotype restored. Involved DNA repair mechanisms are, besides the homology directed repair (HDR) and the non-homologous end-joining (NHEJ), especially the mismatch mediated repair (MMR) pathway with the key players EXO1, MSH3, and PCNA. Chondrosarcoma cells regenerates the majority of DNA damages within 24 h. These molecular mechanisms represent an important basis for an improved therapy.

## Introduction

Chondrosarcoma constitute a heterogeneous group of primary malignant bone tumors with an incidence of 1:50,000 and represent the second most common primary malignant bone tumor^1,2^. Resistance to chemo- and radiotherapy is a consequence of the underlying phenotype, which includes poor vascularization, slow division rate, and hyaline cartilage matrix that prevents access to the cells. Although surgery remains the initial treatment standard, radiation therapy is an important treatment option for chondrosarcoma. The delivery of high radiation doses is challenged by adjacent radiosensitive organs at risk^3,4^.

Proton therapy is advantageous compared to photon therapy due to their superior physical properties, i.e. their major energy deposition at the end of their range and their higher ionization densities. The biological characteristics are less damage to healthy tissue, as well as altered DNA damage and cellular signaling which are reportedly depending on the ionisation density pattern (= linear energy transfer LET)^5–8^. To date, there is a lack of (large) randomized prospective proton therapy related studies for chondrosarcomas. This might be explained on the one hand by the fact that it is a rare tumour and on the other hand by the fact that particle therapy is only available in a few dedicated facilities centres.

Clinical proton therapy is still based on the radiobiological concept of a constant relative biological effective value (RBE) of 1.1 compared to photon beams. The validity of this concept, which was introduced more than five decades ago, is controversially discussed^9,10^. Moreover, there is a general knowledge gap in the fundamentally important field of radiobiology at the molecular level in proton therapy.

As far as chondrosarcomas are concerned, it is imperative to strive for better treatment options for unresectable or metastatic disease. Irreparable DNA double-strand breaks (DSBs) are the main reason for irradiation (IR) induced cell death. Both photon and proton radiation are defined as low linear energy transfer (LET) radiation, but have a distinct different LET distribution. Consequently, DNA damage caused by clinically relevant proton and photon radiation may be different and require different DNA repair capacities^9^. Deregulated signaling networks that control cellular processes such as survival, proliferation and metastasis interact in the radiation resistance of tumor cells. These cellular responses include the DNA damage response induced by ionising radiation^11,12^.

The underlying cellular processes associated with photon and proton IR need to be further investigated and understood in chondrosarcomas. In this context, it is important that the experimental settings mimic clinical IR conditions, as many radiobiological phenomena are proton energy and consequently LET dependent. Several published studies have been performed under experimental conditions with rather low proton energies and high LET values that do not reflect clinical IR conditions^11,12^. Our study focused on the basic cellular processes of chondrosarcoma cells after proton IR in a clinically relevant proton energy range, including a comparison to photon IR. More specifically, we go beyond viability and proliferation behavior and also analyse cell cycle distribution, DNA damage and related DNA repair mechanisms, and changes in the metabolism of irradiated cells. To our knowledge, this is the first study aiming at a comprehensive characterization of the radiation response of human chondrosarcoma cells.

## Results

### Both photon and proton IR alter the proliferation and cell cycle of chondrosarcoma cells

**Figure 1:**
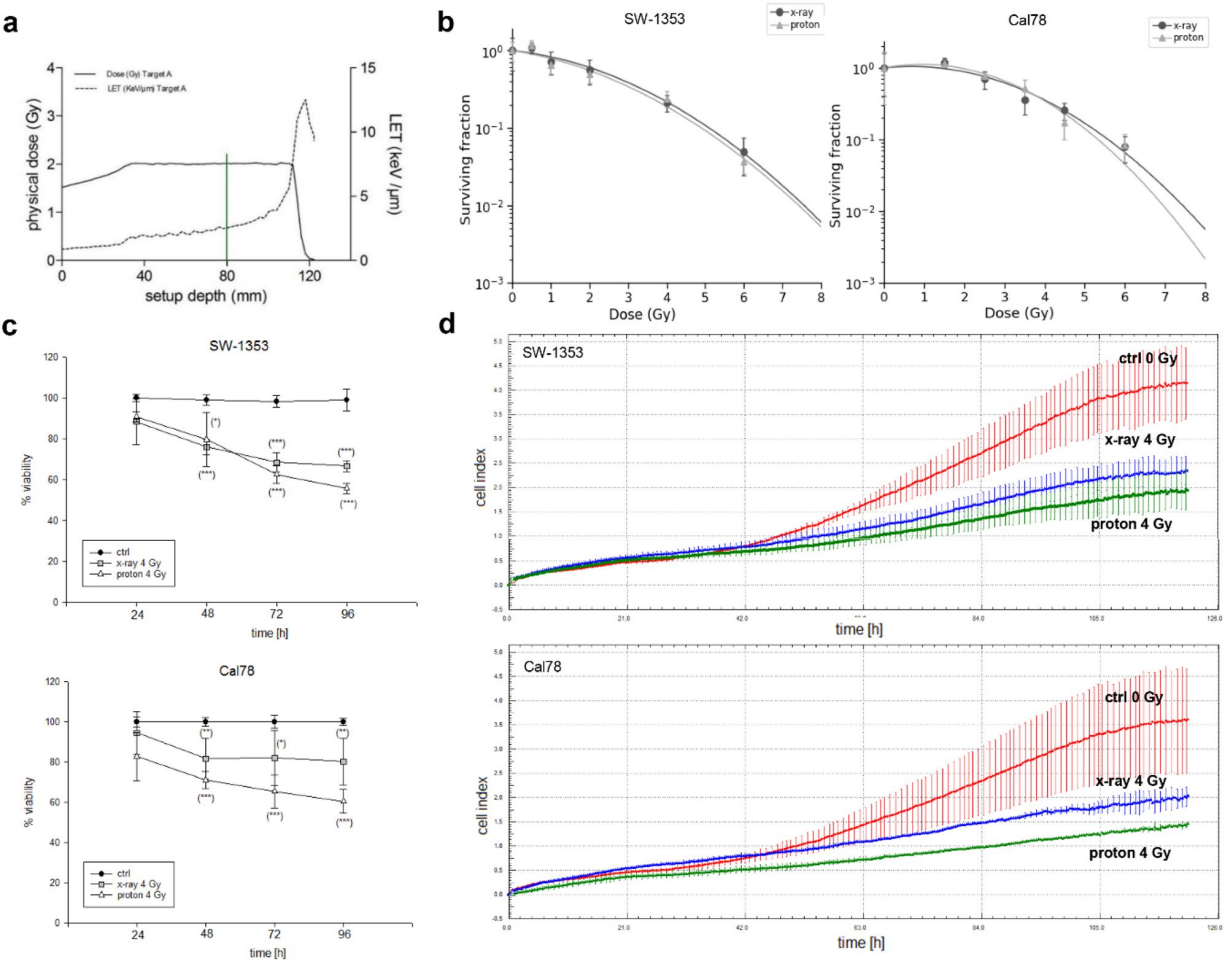
Clonogenic survival, viability and proliferation analysis after photon and proton IR. (**a**) The LETd is based on Monte Carlo calculations and was derived directly from the treatment planning system. (**b**) For clonogenic survival assay SW-1353 and Cal78 cells were harvested immediately after 0.5, 1, 2, 4, and 6 Gy photon (X-ray) and proton IR and seeded according the dose level. Colonies were stained with crystal violet and the surviving fractions were analysed. IR inhibits the ability of chondrosarcoma cells to form colonies dose-dependent manner (mean ± SD; n = 4; measured in sixfold determination). (**c**) Viability and (**d**) xCELLigence real-time proliferation analysis revealed minor differences between the two types of IR on both cell lines (red: unirratiated control cells; blue: X-ray 4 Gy; green: proton 4 Gy).

The dose-averaged linear energy transfer (LET_d_) of all radiation experiments is based on Monte Carlo calculations and was derived directly from the treatment planning system (Fig. 1a). Clonogenic survival assays were performed after 0 (ctrl), 1, 2, 4, and 6 Gy of photon or proton IR; their corresponding surviving fractions are presented in Fig. 1b. Chondrosarcoma cells lost their ability to form colonies with increasing IR doses. The relative biological effectiveness (RBE) at 10% of cell survival was calculated to be 1.04 ± 0.06 Gy for SW-1353 and 1.05 ± 0.07 Gy for Cal78, respectively. Analysis of viability with end time measurements between 24 and 168 h (Fig. 1c) and proliferation of chondrosarcoma cells measured with the real-time xCELLigence system (Fig. 1d) showed no significant differences between the effects of photon and proton IR. Since both cell lines respond similary to IR regarding clonogenicity, viability and proliferation, we focused on the SW-1353 cell line for all further experiments due to restricted proton beam time availability.

**Figure 2:**
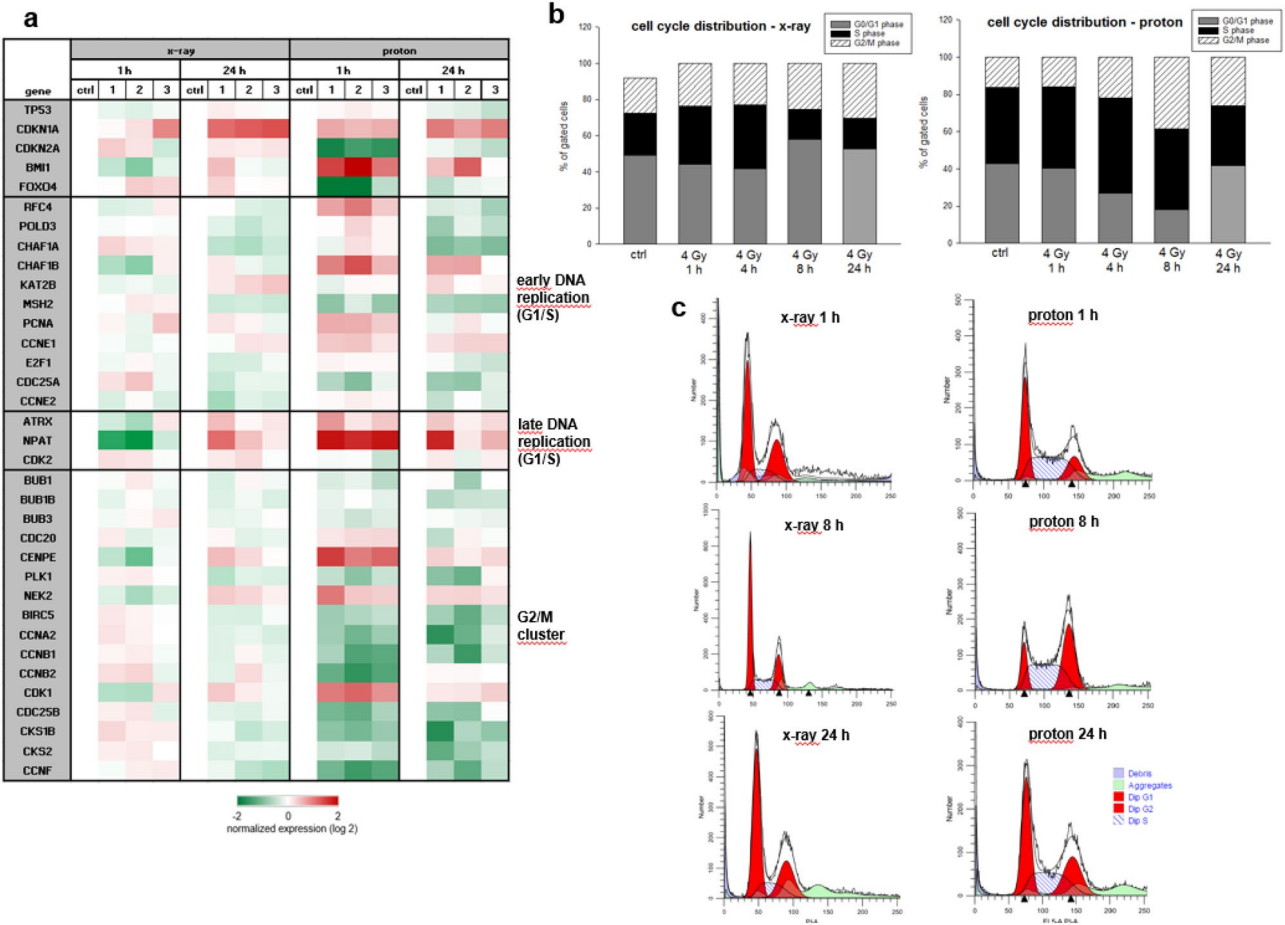
Cell cycle analysis. (**a**) Heatmap blot of RNA sequencing data of relevant cell cycle regulator genes presented in log2 fold‑change after photon (X-ray 6 Gy; left) and proton (4 Gy; right) IR (n = 3). (**b**) Cells were analysed using flow cytometry 1 h, 4 h, 8 h, and 24 h after X-ray or proton IR. The corresponding statistical evaluation shown in stacked bar charts (n = 5). 4 h and 8 h after proton IR the cells are clearly arrested in the G2/M phase, whereas after 24 h this effect is largely reversed. X-ray irradiated cells showed hardly any effects. (**c**) Representative original tracks of non-IR control cells (ctrl) and the photon X-ray respectively proton irradiated samples (4 Gy) are shown.

In the context of the altered cell cycle, we analysed the most important genes of different cell cycle phases using RNA expression profiling 1 h and 24 h after photon and proton IR. Heatmap plot of RNA sequencing data were presented in log2 transformed fold‑change regarding expression of cell cycle regulation genes alterations after IR (Fig. 2a). Similar regulation can be observed with both types of IR, whereby the proton IR produces significantly stronger effects. Especially 1 h after proton IR, the available data showed an upregulation of genes involved in both, cell cycle regulation (CDKN1A, NPAT, CENPE, NEK2, CDK1) and DNA repair (BMI1, ATXR). To investigate the effects on the cell cycle, 1 h, 4 h, 8 h, and 24 h after proton IR cells were analyzed using flow cytometry. While changes in the cell cycle after X-ray IR are only minor, significant differences were observed after proton irradiation. Proton IR caused a decrease in the number of cells in the G1 phase (black bars) and S phase (striated bars), accompanied by a significant increase of the number of G2/M phase (grey bars) cells, indicating a G2/M arrest (Fig. 2b). Cell cycle changes were found to be time dependent, with marginal effects at 1 h and pronounced effects at 4 h and 8 h post-proton IR. Within a period of 24 h, however, the tumor cells regenerate almost completely. Representative measurements of non-IR (ctrl) and IR cells are depicted to highlight the differences (Fig. 2c). All values of five individual X-ray and proton IR experiments (% of gated cells) and their statistical differences are listed in Table 1.

**Table 1:** Cell cycle distribution of chondrosarcoma cells 1 h, 4 h, 8 h, and 24 h after 4 Gy X-ray respectively proton IR (n = 5; mean ± SD; *n.s.* not significant).

	G0/G1 (%)	S (%)	G2/M (%)
ctrl X-ray	49.23 ± 3.77	23.25 ± 6.08	19.17 ± 3.05
ctrl proton	42.91 ± 1.27	40.90 ± 0.43	16.19 ± 1.01
4 Gy X-ray 1 h	44.15 ± 12.65	31.99 ± 9.14	23.85 ± 4.19
	n.s.	n.s.	n.s.
4 Gy proton 1 h	40.30 ± 1.94	43.87 ± 1.99	15.86 ± 2.23
	n.s.	n.s.	n.s.
4 Gy X-ray 4 h	41.66 ± 12.24	35.29 ± 11.89	23.04 ± 1.10
	n.s.	n.s.	n.s.
4 Gy proton 4 h	26.95 ± 2.83	51.11 ± 1.66	21.94 ± 1.92
	p = 0.0003	p = 0.0001	n.s.
4 Gy X-ray 8 h	57.97 ± 9.64	16.56 ± 4.62	25.47 ± 10.09
	n.s.	n.s.	n.s.
4 Gy proton 8 h	17.95 ± 3.55	43.57 ± 2.46	38.48 ± 1.95
	p < 0.0001	n.s	p < 0.0001
4 Gy X-ray 24 h	52.50 ± 13.53	17.12 ± 5.82	30.38 ± 9.15
	n.s.	n.s.	p = 0.029
4 Gy proton 24 h	41.55 ± 0.56	32.28 ± 1.24	26.10 ± 1.60
	p < 0.0001	p = 0.0002	p = 0.002

### Double strand breaks are repaired by chondrosarcoma cells within 24 h

**Figure 3:**
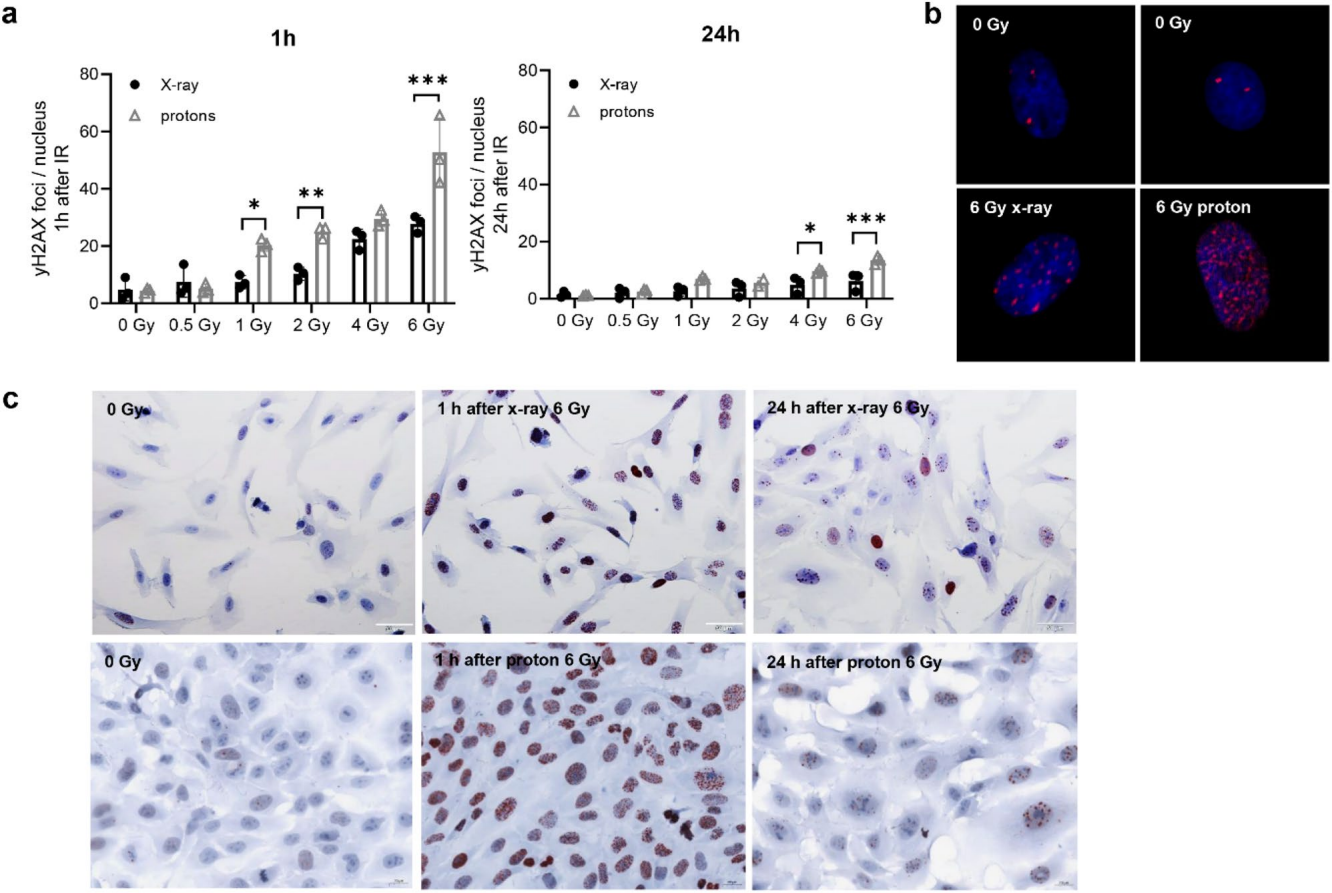
Detection of the DNA damage marker γH2AX. Chondrosarcoma cells were irradiated with 0.5, 1, 2, 4, and 6 Gy photon (X-ray) respectively proton ion particles and analysed using immunofluorescence and immunohistochemical stainings. (**a**) The statistical evaluation over the course of the IR dose. (**b**) Representative pictures of stained γH2AX foci in a single nucleus. (**c**) Immunohistochemical staining of γH2AX 1 h and 24 h after IR (n = 3; × 40 magnification). The significant increase of γH2AX observed after 1 h is hardly observed after 24 h.

The amount of double strand breaks was determined by quantifying γH2AX foci 1 and 24 h after 0–6 Gy photon respectively proton IR. Representative immunofluorescence staining of foci/cell 1 h after IR were displayed in Fig. 3a. Chondrosarcoma cells showed a dose-dependent increase in foci 1 h after proton IR for all doses higher than 0.5 Gy, whereas after 24 h only minor residual DNA damage was observed (Fig. 3b). These observations could be confirmed by immunohistochemical staining (Fig. 3c). At 1 h post radiation, significantly more γH2AX foci after protons as compared to X-rays were observed for 1 Gy (p = 0.0167), 2 Gy (p = 0.005) and 6 Gy (p < 0.0001). After 24 h significant differences were noted for 4 Gy (p = 0.0261) and 6 Gy (p = 0.003).

### Proton irradiation efficiently activate DNA repair mechanisms

**Figure 4:**
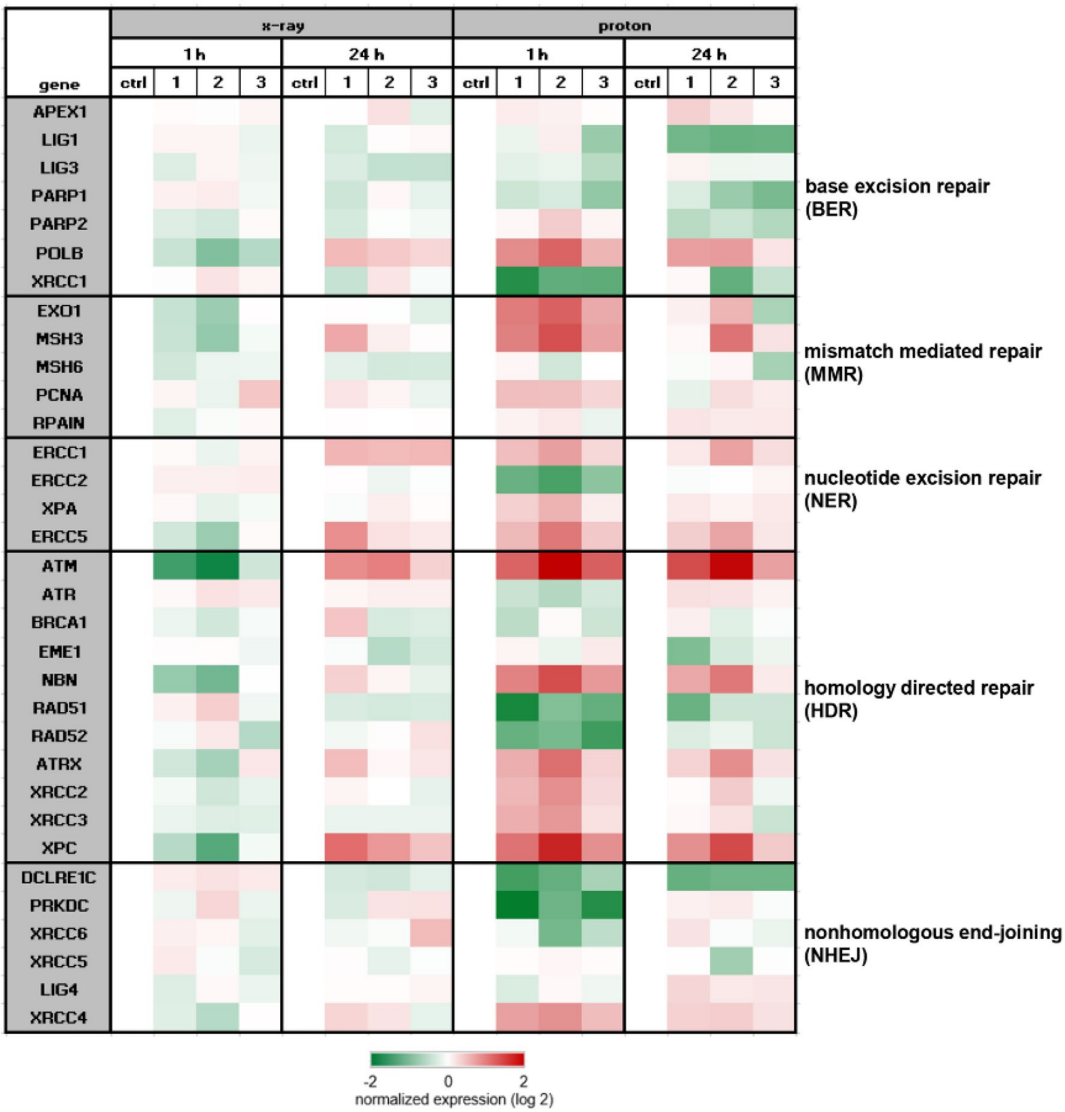
Next generation sequencing data. Heatmap blot of RNA sequencing data of the key players of DNA repair mechanism pathways presented in log2 fold change (n = 3) 1 h and 24 h after 6 Gy photon (X-ray; left) and 4 Gy proton IR (right).

To investigate the importance of the different DNA repair mechanisms, we isolated RNA 1 h and 24 h after IR and performed RNA expression profiling. Heatmap plot of RNA sequencing data was presented in log2 transformed fold‑change regarding expression without (ctrl) and 1 h and 24 h after 4 Gy photon or proton IR of key player genes of the base excision repair (BER), the mismatch mediated repair (MMR), the nucleotide excision repair (NER), the homology directed repair (HDR), and the non-homologous end-joining (NHEJ) (Fig. 4). The course is very similar with both types of IR, whereby the regulations are considerably more pronounced with the proton IR. It was revealed that not only the HDR pathway, known from literature, is positively regulated, but also the MMR and NER pathway.

**Figure 5:**
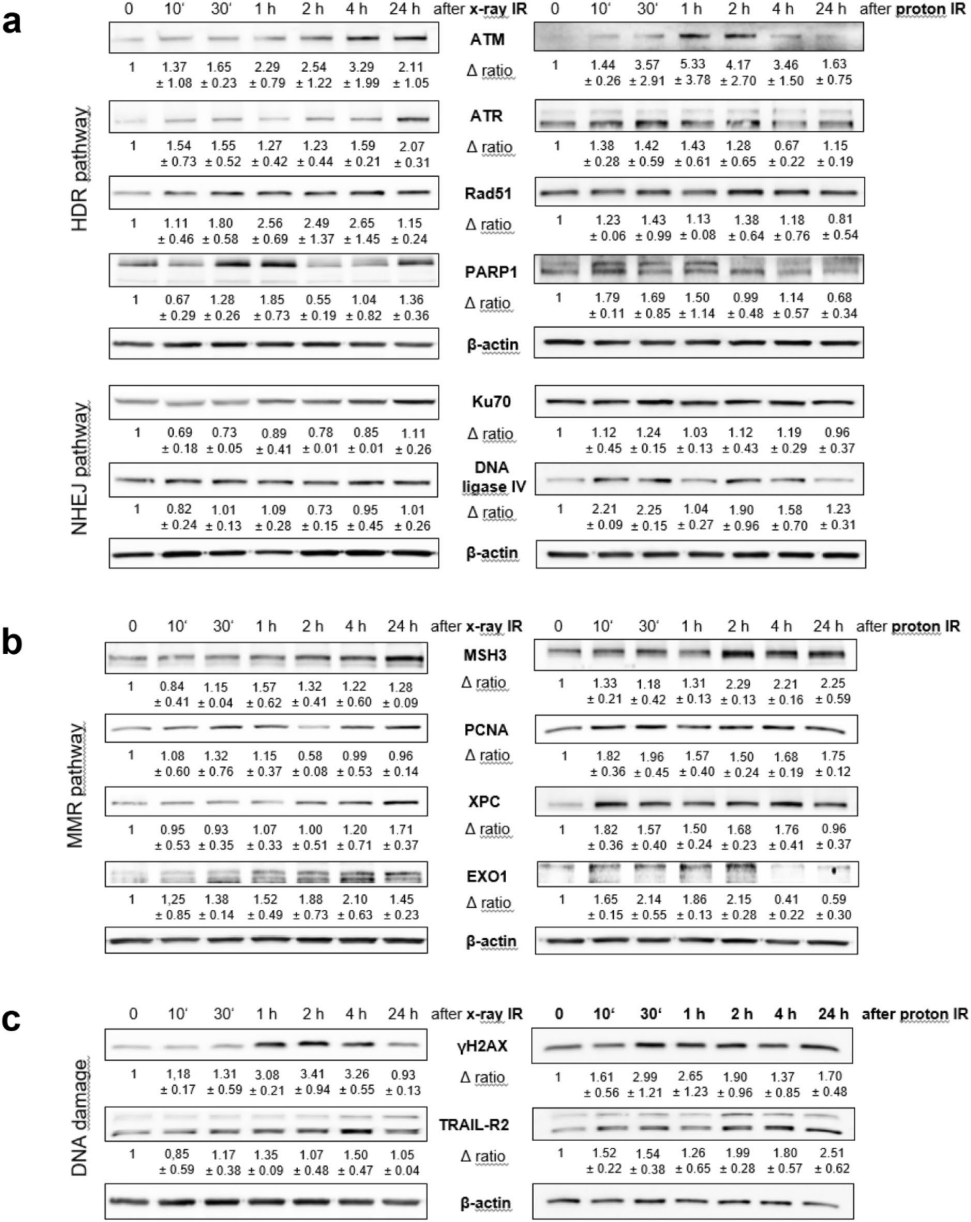
DNA damage. Protein expression of the most important regulators of (**a**) the homology directed repair (HDR) and the non-homologous end-joining (NHEJ) pathways, (**b**) the mismatch mediated repair (MMR) pathway, and (**c**) the DNA damage marker γH2AX and the death receptor TRAIL-R2. The individual key players were evaluated by immunoblotting under control conditions (0 Gy) and 10 min, 30 min, 1 h, 2 h, 4 h, and 24 h after photon (X-ray) or proton IR. β-actin was used as loading control. Δ ratio, fold change normalized to non-IR controls (mean ± SD of n = 4). Full-length blots/gels are presented in Supplementary Fig. S1.

Corresponding to the RNA sequencing data the expression of ATM protein increased fivefold after 1 h and 2 h proton IR. Afterwards the expression decreased. A 1.5–2.5-fold increase of the ATR, Rad51, PARP1, and DNA ligase IV expression occurred already after a few minutes, while Ku70 remained unchanged (Fig. 5a, right). Very similar effects were observed after X-ray IR. However, the changes in protein expression occurred with a time delay (Fig. 5a, left). In accordance with the RNA sequencing data, the protein expression of MSH3, PCNA, XPC, and EXO1 increased within a very short time as a result of proton IR (Fig. 5b, right). This pathway was also activated significantly later over time by the X-ray IR (Fig. 5b, left). The phosphorylated histone γH2AX was elevated threefold in response to proton IR already after 30 min (Fig. 5c, right), and after 1–2 h with X-ray IR (Fig. 5c, left). The protein expression data confirmed the IF and IHC findings, for instance that DNA damage is repaired largely within 24 h in chondrosarcoma cells.

**Figure 6:**
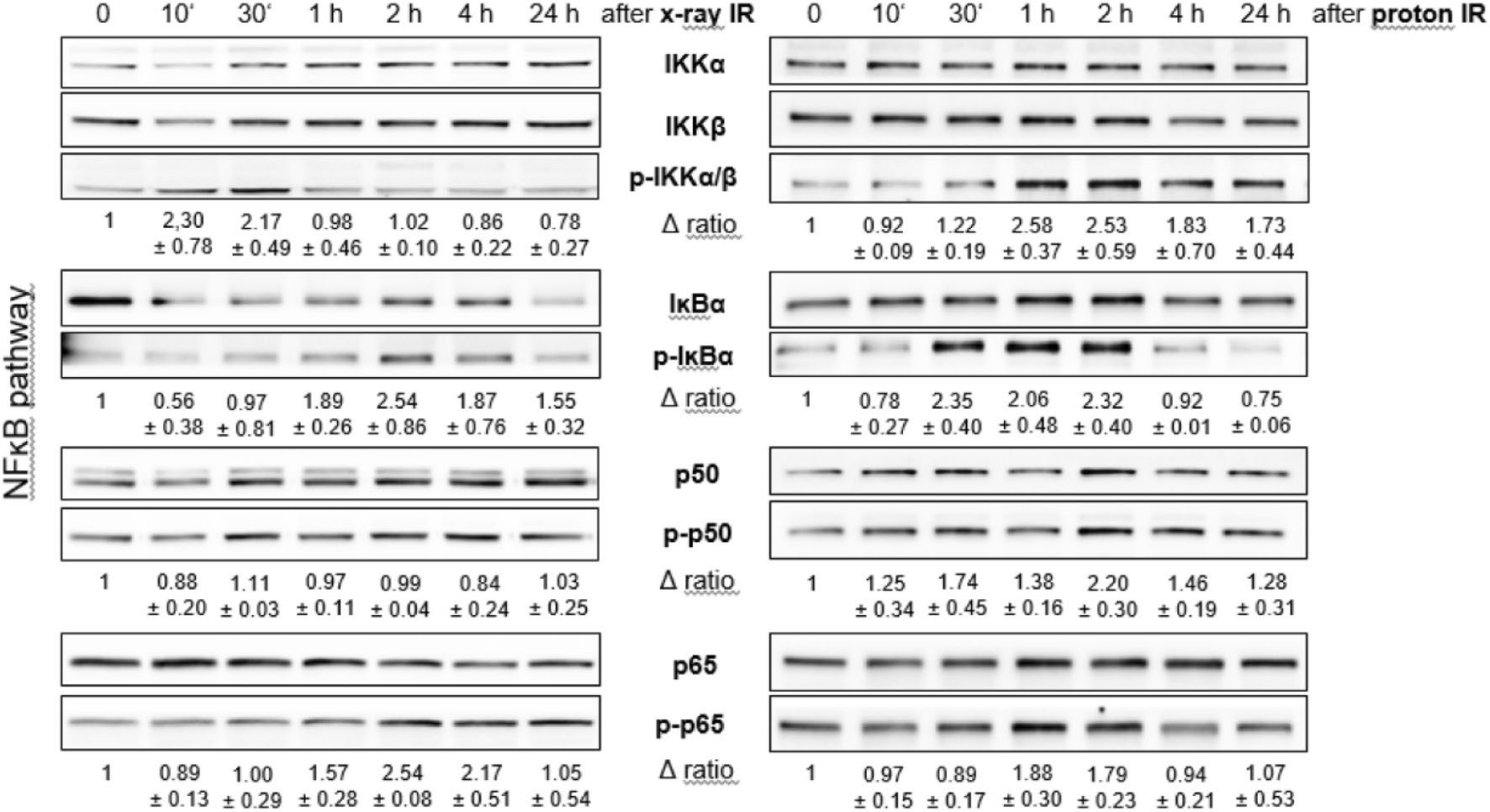
NF-ĸB damage pathway. Protein expression and phosphorylation of components of the NF-ĸB pathways were evaluated by immunoblotting under control conditions (0 Gy) and 10 min, 30 min, 1 h, 2 h, 4 h, and 24 h after photon (X-ray) or proton IR. β-actin was used as loading control. Δ ratio, fold change normalized to controls (mean ± SD of n = 4). Full-length blots/gels are presented in Supplementary Fig. S1.

The time-delayed 2.5-fold increase within the first four hours in the expression of the death receptor TRAIL-R2 proved the damage of the chondrosarcoma cells. Proton IR activates the NF-ĸB pathway in chondrosarcoma cells. Whole cell lysates were extracted from the cells 10 min, 30 min, 1 h, 2 h, 4 h, and 24 h after 4 Gy proton IR and prepared for western blot analysis. Fold changes normalized to non-IR controls (Δ ratio; mean ± SD of n = 3) were presented. 30 to 60 min after proton IR there is an increased phosphorylation of IKKα/β (2.6-fold), IĸBα (2.3-fold), p52 (1.7-fold), and p65 (1.8-fold). In this pathway, too, X-ray IR caused a later activation than proton IR (Fig. 6).

### Metabolic phenotyping

**Figure 7:**
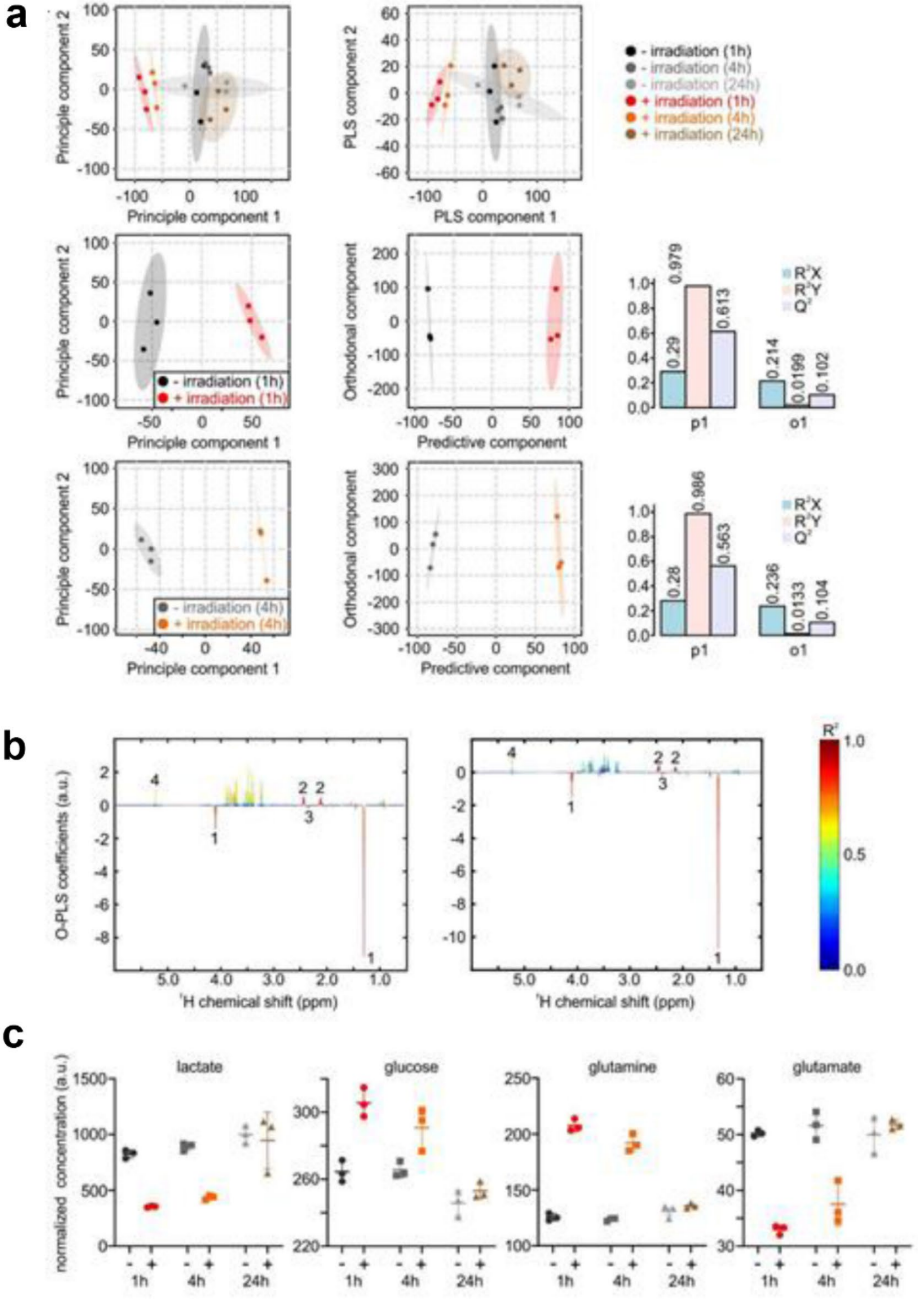
Effect of proton irradiation on the metabolic activity of chondrosarcoma cells. (**a**) Principal Component Analysis (PCA) and (Orthogonal) Partial Least Squares Discriminant Analysis ((O)-PLS-DA) of cell culture supernatants 1 h, 4 h, and 24 h after 4 Gy proton IR versus non-IR control conditions (n = 3). (**b**) Reduced NMR spectra reveal altered metabolites in normalized cell culture supernatants at 1 h (left) and 4 h (right). Positive covariance corresponds to metabolites present at increased concentrations, whereas negative covariance corresponds to decreased metabolite concentrations in supernatants of irradiated samples compared to the corresponding control samples. Predictivity of the model is represented by R2. 1…lactate, 2…glutamine, 3…glutamate, 4…glucose. (**c**) shows the alteration in the normalized concentrations of lactate, glucose, glutamine, and glutamate without (−) and with (+) 4 Gy proton IR.

In order to assess metabolic differences between control cells and photon respectively proton irradiated cells, nuclear magnetic resonance (NMR) metabolic profiling of three independent experiments was performed. After photon IR, there were no significant differences in metabolic activity between the unirradiated controls and the irradiated cells (data not shown). Proton IR caused a strong shift of the metabolic profiles of cell supernatants, as indicated by the discriminant clustering observed in the Principal Component Analysis (PCA) and Partial Least Squares Discriminant Analysis (PLS-DA) plots (Fig. 7a top). The differences in metabolic profiles were largest 1 h after IR and shifted back to the metabolic profiles observed for non-irradiated cells within 24 h. The discriminant clustering 1, and 4 h after IR, respectively shown in the orthogonal-partial least squares-discriminant analysis (O-PLS-DA) plot in Fig. 7a indicates the underlying differences in the metabolome, supported by the correlation coefficients R^2^Y up to 0.986 and a positive Q^2^ of 0.683, validating the significance of these results. Reduced NMR spectra revealed altered levels of metabolites in normalized cell culture supernatant samples and indicated that the levels of lactate (Lac) and alanine (Ala) were diminished, whereas concentrations of glucose, glutamine, and branched-chain amino acids (BCAAs) like valin, leucin, and isoleucin were higher 1 h after proton IR (Fig. 7b). In addition, the succinate and glutamate levels remained reduced 4 h after IR (Fig. 7c). Remarkably, and in agreement with the cell cycle and γH2AX data, this indicates recovery of metabolic rearrangement under proton IR.

## Discussion

Particle therapy with protons or heavier ions is one of the most advanced forms of radiotherapy and offers new opportunities for improvements in cancer care^13,14^. Proton therapy for chondrosarcomas is advantageous compared to photon therapy and some clinical studies reported favourable local control, survival, and toxicity^15–18^. Radiation resistance remains a major obstacle, which limits the effectiveness of radiation therapy. However, cellular and molecular processes in chrondrosarcoma cells are hardly known and little experimental data is available in literature. In order to improve the efficacy of radiotherapy, it is essential that we fully understand the signaling network that causes cancer cells to overcome radiation-induced cytotoxicity, which was the main motivation for this study.

In order to ensure clinical relevance, chondrosarcoma cells were irradiated with clinically relevant energies of a proton beam and corresponding photon doses. Even though the viability of the cells only marginally decreases, the cells lost their ability to form colonies with increasing dose. We assume that the reason for reduced colonies despite only marginally reduced viability is an inherent unsuitability of the colony forming assay for chondrosarcoma cells. This was observed with both investigated chondrosarcoma cell lines. Further indication of colony forming assay inadequacy is illustrated by RBE values being similar after photons and protons despite observable differences for all other endpoints investigated. Due to rapid growth, especially of unirradiated control cells and at lower doses, the colony forming assay had to be stopped after a relatively short time periods of 5 or 9 days for SW-1353 or Cal78, respectively, in order to facilitate colony identification. We speculate that time needed for the observed extensive repair results in delayed growth and hence fewer colonies in samples irradiated with higher doses, despite viability remains high.

Chondrosarcoma cells appear to display a similar response compared to other cancer cells, depicted by significantly altered cell cycles and DNA damage repair following protons^19,20^. As a result of DNA damage, cell cycle control points are activated which block the cell cycle to allow the cell to repair^21,22^. ATM and ATR kinases are rapidly activated, which leads to the activation of downstream targets such as p53, Chk1 and Chk2 kinases and may directly inhibit the activity of the CDK1/cyclin B complex^23^. Our RNA expression profile of cell cycle-relevant genes showed exactly these signal transduction cascades: an enhanced expression of p53 (TP53), CDK1, and p21 (CDKN1A) 1 h after 4 Gy proton IR and a prolonged inhibition of CDC25 and cyclin B2 (CCNB2). It has been shown that cyclin B1/CDK1 functions in the communication between mitochondrial activity and cell cycle progression^24^. CDK1 is involved in the integration of mitochondrial fission during G2/M transition and stimulates mitochondrial ATP production to meet the increased energy requirements for DNA repair and cell survival^25,26^. As a result, a significant G2/M arrest occurs 4 h and 8 h after IR. Flow cytometry analysis clearly showed that this arrest regressed after 24 h, indicating a high DNA repair efficacy.

The sustained localization of BMI1 to sites of DNA damage is dependent upon ATR/ATM and H2AX phosphorylation^27^. Further, the increase in NPAT after proton IR is remarkable, which is required for progression through the G1 and S phases of the cell cycle, activates transcription of the histone genes, and positively regulates the ATM promoter^28^. Radiation induced cell death is mostly due to DNA damage, especially to double-strand breaks^29^. H2AX foci specifically attract DNA repair factors, resulting in an accumulation of DNA damage signalling and repair proteins around a DNA double-strand break^30^. Specific recognition of H2AX by these repair factors requires the presence of protein domains that bind to the phosphorylated carboxy terminus of H2AX. γH2AX phosphorylation induced 1 h after IR was clearly shown by immunofluorescence and immunohistochemistry as well as on protein levels. The rapid regeneration potential of chondrosarcoma cells is particularly clearly visible at 24 h after treatment, when γH2AX phosphorylation was already mostly resolved.

With regard to a Rad51 expression, Venneker et al. achieved similar results^31^. After a 2 h recovery of γ-radiation treatment, chondrosarcoma cell lines showed a significant induction of RAD51 foci, indicative of a proficient homologous recombination pathway. However, 24 h after IR, CH2879 and SW-1353 cells each exhibited evidence of recovery as reflected by partial repair of DNA damage, while JJ012 cells retained DNA damage signals. Supplementary to the HDR and NHEJ pathways, described in the literature for other cancer entities^32–36^, the MMR pathway with the key players EXO1, MSH3, and PCNA, is also clearly activated in chondrosarcoma cells after proton application. Our data show for the first time the different regulation of DNA repair mechanisms in human chondrosarcoma cells following proton irradiation as compared to photons. The inhibition of these different regulatory mechanisms may offer great potential for improving radiotherapy in chondrosarcoma. Cesaire et al. demonstrated the capacity of the PARP inhibitor Olaparib to radiosensitize chondrosarcoma cells to proton irradiation^37^. Further investigations in this area are of exceptional importance.

Furthermore, ionising radiation activates the transcription factor NF-κB, which is a trigger for the resistance of cancer cells to radiation therapy. Elevated NF-ĸB activity in the presence of irradiation is directly correlated with radiation resistance^38^. 30–60 min after a 4 Gy proton application the members of the pathway showed an increased phosphorylation level. Thus, the interruption in IκB degradation, proteasome action, IKK phosphorylation, and NF-κB nuclear translocation provide promising therapy strategies for inhibiting adverse effect of proton-induced NF-κB activation, which would be worth investigating.

In order to investigate the basic metabolic processes in the irradiated cells, we carried out a so-called metabolic phenotyping using NMR spectrometry. These data revealed a reduced consumption of glutamine and glucose, along with the reduced secretion of lactate compared to control cells without IR indicates that chondrosarcoma cells become less metabolically active upon proton IR. This is in agreement with the induction of a senescent state with reduced proliferation, which is in line with the increased levels of FOXO4 previously observed in senescent cells^39^.

Although viability and cell proliferation decreased in a very similar way with both types of IR, stronger gene expression regulation was generally observed after proton IR. Both the IR-induced cell cycle G2/M arrest and the γH2AX phosporylation almost returned to the level of the non-IR control group after 24 h. Analyses of DNA repair genes revealed, in besides the HDR and NHEJ pathway, also an activation of the MMR pathway. In order to further improve the therapeutic success, the inhibition of the cell's own DNA repair mechanisms will be of outstanding importance. This could be reached by charged particles like carbon ions, which will be focus of the next experiments.

## Methods

### Cell culture

SW-1353 (RRID: CVCL_0543) chondrosarcoma cell line (ATCC^®^ HTB-94™, LGC Standards, Middlesex, UK) and Cal78 (ACC459, DSMZ, Leibnitz, Germany) were cultured in Dulbecco’s-modified Eagle’s medium (DMEM-HG) supplemented with 10% FBS, 1% l-glutamine, 1% penicillin/streptomycin, and 0.25 µg amphotericin B (all GIBCO^®^, Invitrogen, Darmstadt, Germany). The cell line was authenticated by STR profiling within the last three years. All experiments were performed with mycoplasma-free cells. In order to be able to better classify the gene expression data, we point out that the cell line SW-1353 has a TP53 and MDM2 mutation.

### Photon and proton IR set up

Photon (X-ray) IR was performed at the Division of Biomedical Research, Medical University Graz (Graz, Austria) with an RS-2000 biological irradiator (RadSource Technologies, Inc., Buford, GA, USA) equipped with a 3-mm Al/0.3-mm Cu filter and a current of 25 mA to provide 160 kV X-rays at a dose rate of 2.1 Gy/min. Proton IR was performed at the synchrotron-based Austrian center for ion therapy and research (MedAustron)^40^, which is equipped with a horizontal beam line including an active spot scanning technique with active energy variation for proton ions. The exact and standardized positioning of the samples was performed by a high precision robot couch and a laser positioning system in three dimensions. To facilitate positioning of biological samples, a phantom consisting of PMMA (Polymethylmethacrylat) was designed^41^. A clinically relevant target size, requiring proton energies between 66.5 and 135.6 MeV, which translated into a dose-averaged linear energy transfer (LETd) of 2.8 keV/µm, was selected for our study. The LETd is based on Monte Carlo calculations and was derived directly from the treatment planning system.

### Colony forming units

Cells were harvested immediately after IR and were seeded on 6-well dishes in concentrations according the dose level. Following a 5 days (SW-1353) respectively 9 days (Cal78) incubation period, colonies were fixed with 96% methanol and stained with 0.5% crystal violet solution. A minimum of 50 cells were considered as a colony. Based on the linear quadratic model, surviving fractions in reference to the plating efficiency of non-IR control samples were calculated for each delivered physical dose (in Gy) (n = 4; in sixfold determination).

### Viability and proliferation analysis

For the dose–response relationship, SW-1353 cells were irradiated with 0 Gy (neg. control) and 4 Gy photon respectively proton IR. Cell viability was determined with the CellTiter-Glo^®^ cell viability assay (Promega Corporation, Madison, MI, USA) after 24–168 h and normalized to the non-IR controls. Background reference values were derived from the culture media. Absorbance was measured with a LUMIstar™ microplate luminometer (BMG Labtech GmbH, Ortenberg, Germany) (mean ± SD; n = 7, performed in biological quadruplicates). The xCELLigence RTCA-DP device (OLS, Bremen, Germany) was used to monitor cell proliferation in real-time. Cells were seeded after IR in electronic microtiter plates (E-Plate™, OLS) and measured for five days according to the manufacturer´s instructions. Cell density was measured in quadruplicate with a programmed signal detection every 20 min. Data acquisition and analyses were performed with RTCA software (version 1.2, OLS).

### Cell cycle analysis

1 h, 4 h, 8 h, and 24 h after 4 Gy photon or proton IR cells were harvested by trypsinization and fixed with 70% ice-cold ethanol for 10 min at 4 °C. Before flow cytometry analysis, the cell pellet was resuspended in propidium iodide (PI)-staining buffer (50 μl/ml PI, RNAse A) and incubated for 15 min at 37 °C. Cell cycle distribution was measured with CytoFlexLX (Beckman Coulter, Pasadena, CA, USA) and analyzed using ModFit LT software Version 4.1.7 (Verity software house). Four independent experiments were conducted in each case.

### Immunohistochemistry and immunofluorescence staining

For immunohistochemical staining, cells irradiated with doses of 0 (control) and 6 Gy were fixed after 1 h and 24 h with 4% paraformaldehyde for 30 min. Slides were incubated with γH2AX antibody (Merck, Darmstadt, Germany) for 1 h, the bridge antibody (Dako Agilent, Jena, Germany) for 30 min, the polymer (rabbit-ON-rodent-horse radish peroxidases; Biocare Medical, Pacheco, CA) for 30 min, and AEC substrate chromogen (Dako) for 3 min. The reaction was stopped with PBS and then a haemalaun core staining was performed. Pictures were taken with an Olympus BX51 microscope (Olympus, Vienna, Austria). For immunofluorescence imaging cells irradiated with doses of 0 (control), 0.5, 1, 2, 4, 6 Gy. The fixation was performed after 1 h and 24 h with 4% paraformaldehyde for 30 min. After incubation with the permeabilization solution (PBS/Triton X 0.1%/SDS 0.1%) for 6 min, the blocking solution (PBS/goat serum/BSA 2%) for 30 min at 37 °C, and γH2AX antibody (Merck) for 60 min at room temperature, the secondary antibody (Rhodamine (TRITC)-conjugated AffiniPure Goat Anti-Mouse IgG; Jackson Immuno Research Laboratories, West Grove, PA) was applied. Microscopic evaluation was performed with a Zeiss Imager Z.2 microscope (Zeiss, Oberkochen, Germany) equipped with the Metafer analysis system (MetaSystems, Altlussheim, Germany). Analyses of γH2AX foci was performed using one-way analyses of variance (ANOVA) with post hoc Tukey´s multiple comparison tests. A value of p < 0.05 was regarded statistically significant.

### Gene expression profiling

Gene expression profiling was performed using the Thermo Fisher Ion Ampliseq RNA workflow. Briefly, RNA was transcribed to cDNA using the SuperScript™ VILO™ cDNA Synthesis Kit according to the manufacturer`s protocol. cDNA equivalent to 50 ng RNA was used in a PCR reaction with a custom Ion Ampliseq RNA Panel encompassing amplicons in 69 genes. A next generation sequencing (NGS) Library was generated from the PCR product using the AmpliSeq Library Kit Plus and subsequent library quantification was done using the Ion Library TaqMan™ Quantitation Kit. Sequencing was performed on an Ion S5XL benchtop sequencer using the 540 Chip Kit and the 200 base pair work flow (all Thermo Fisher Scientific, Waltham, MA) to a total depth of approximately one million reads per sample. Individual gene expression is considered to be equivalent to the relative read count of the gene specific amplicon in the total library. Data was analyzed using the Ampliseq RNA Ion Torrent Suite Plugin (version 4.4.0.4) and individual gene expression was calculated as amplicon reads per million total reads (RPM). As Ampliseq RNA is a amplicon counting technology we have reported the number of mapped reads, percent reads on target and percent assigned reads for each sample (n = 3).

### Protein expression analysis

Whole cell protein extracts were prepared with lysis buffer (50 mM Tris–HCl pH 7.4, 150 mM NaCl, 1 mM NaF, 1 mM EDTA, 1% NP-40, 1 mM Na3VO4) and a protease inhibitor cocktail (P8340; Sigma Aldrich), after 10 min, 30 min, 1 h, 2 h, 4 h, 8 h, and 24 h proton IR with 4 Gy. Protein concentration was determined with the Pierce BCA Protein Assay Kit (Thermo Fisher Scientific). The proteins were separated by SDS-PAGE and were blotted on Amersham™ Protran™ Premium 0.45 µM nitrocellulose membranes (GE Healthcare Life Science, Little Chalfont, UK). Primary antibodies against the DNA damage key players ATM, ATR, Rad51, Poly(ADP-ribose) polymerase (PARP)-1, Ku70, DNA ligase IV, phospho-histone γH2AX, TRAIL-R2, and the NF-ĸB pathway components IKKα, IKKβ, phospho IKKα/β, IĸB, phospho IĸB, p50, phospho p50, p65, phospho p65 (Cell Signaling Technology, Danvers, MA) and PCNA, MSH3, EXO1, and β-actin (Abcam, Cambridge, UK) as loading control were used. Blots were developed using a horseradish peroxidase-conjugated secondary antibody (Dako) for 1 h and the Amersham™ ECL™ prime western blotting detection reagent (GE Healthcare). Chemiluminescence signals were detected with the ChemiDocTouch Imaging System (BioRad Laboratories Inc, Hercules, CA) and images were processed with the ImageLab 6.0.1 Software (BioRad Laboratories Inc; www.bio-rad.com/de-at/product/image-lab-software).

### Metabolic phenotyping

Changes in metabolic phenotypes were determined using nuclear magnetic resonance (NMR) spectroscopy (Bruker Topspin, Rheinstetten, Germany). Cell culture supernatants from control and photon respectively proton irradiated samples were lyophilized and 500 µl of NMR buffer (5.56 g Na2HPO4, 0.4 g TSP, 0.2 g NaN3, in 400 ml of D2O; pH 7.4) were added. All NMR experiments were performed at 310 K on an AVANCE™ Neo Bruker Ultrashield 600 MHz spectrometer equipped with a TXI probe head and processed as described previously^42^. The spectra for all samples were automatically processed and referenced using TSP at 0.0 ppm. NMR data were imported to Matlab^®^ vR2014a (Mathworks, Natick, MA), regions around the water, TSP, and remaining MeOH signals excluded, and probabilistic quotient normalization was performed to correct for sample metabolite dilution^43^. To identify changes in metabolic profiles, multivariate statistical analysis was performed as described previously^44^, and includes Principle Component Analysis (PCA), Orthogonal-Partial Least Squares–Discriminant Analysis (O-PLS-DA), and all associated data consistency checks and sevenfold cross-validation. Stated concentrations correspond to normalized concentrations after probabilistic quotient normalization. PCA, PLS-DA and O-PLS-DA figures were prepared using MetaboAnalyst.

## Supplementary Information

Supplementary Figure S1.
