# Supplementary figures and images for: Activation of efficient DNA repair mechanisms after photon and proton irradiation of human chondrosarcoma cells
Source: Sci Rep. 2021 Dec 16;11:24116. doi: 10.1038/s41598-021-03529-9 (PMC8677811; doi:10.1038/s41598-021-03529-9)

uncropped files  
western blot analysis  
Fig.5 and 6

Figure 5a

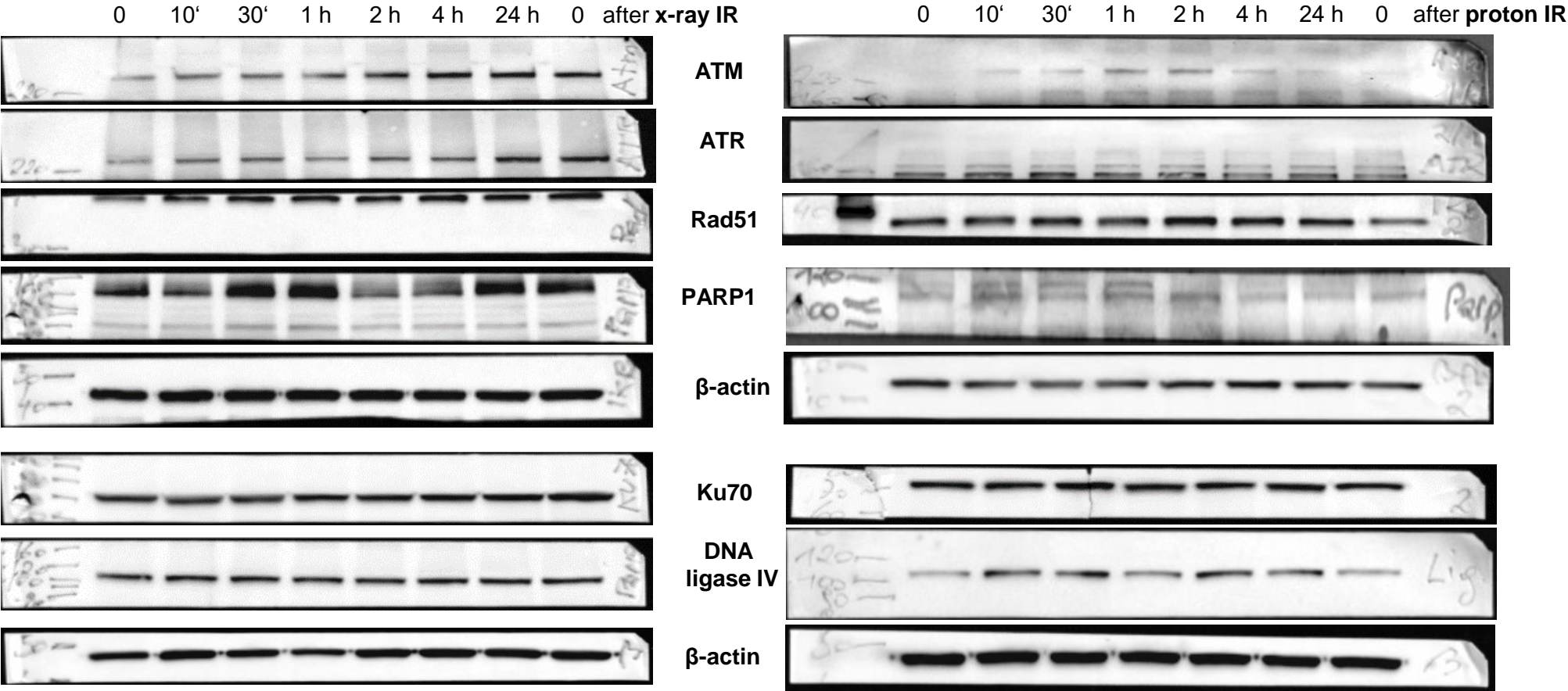

### Figure 5b

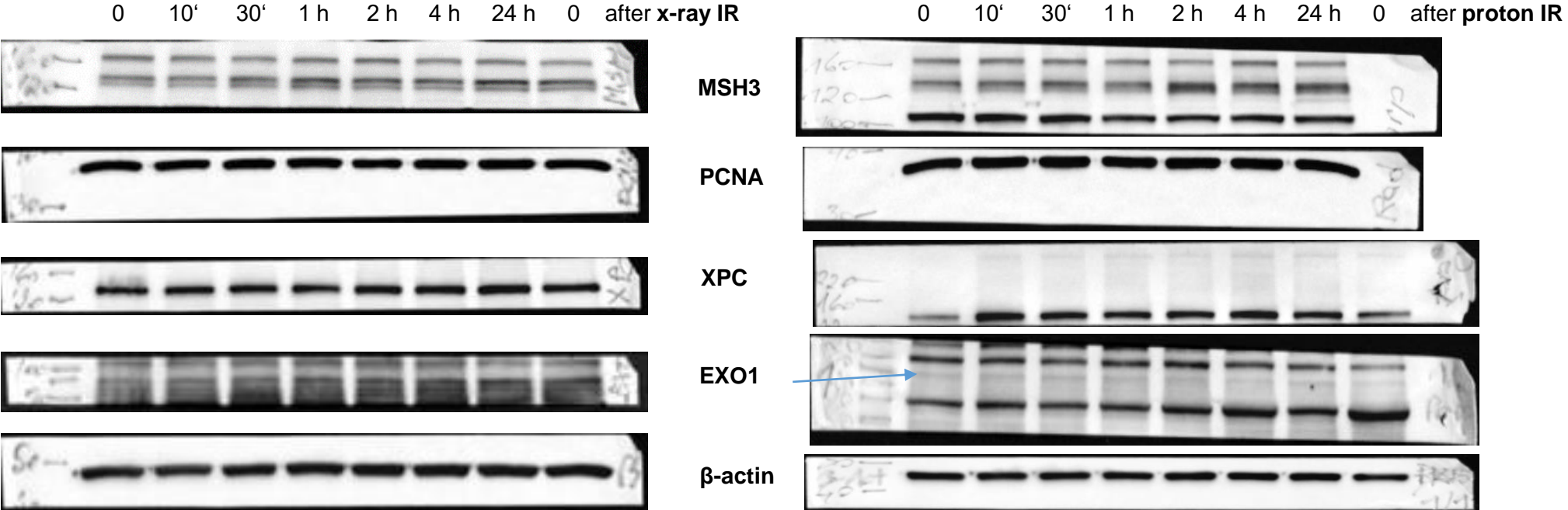

Figure 5c

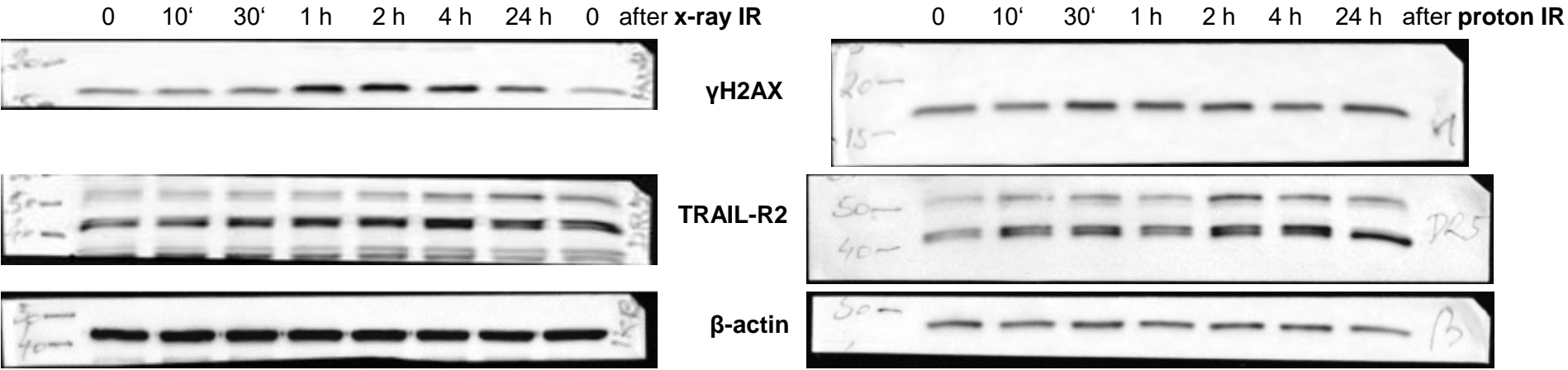

Figure 6

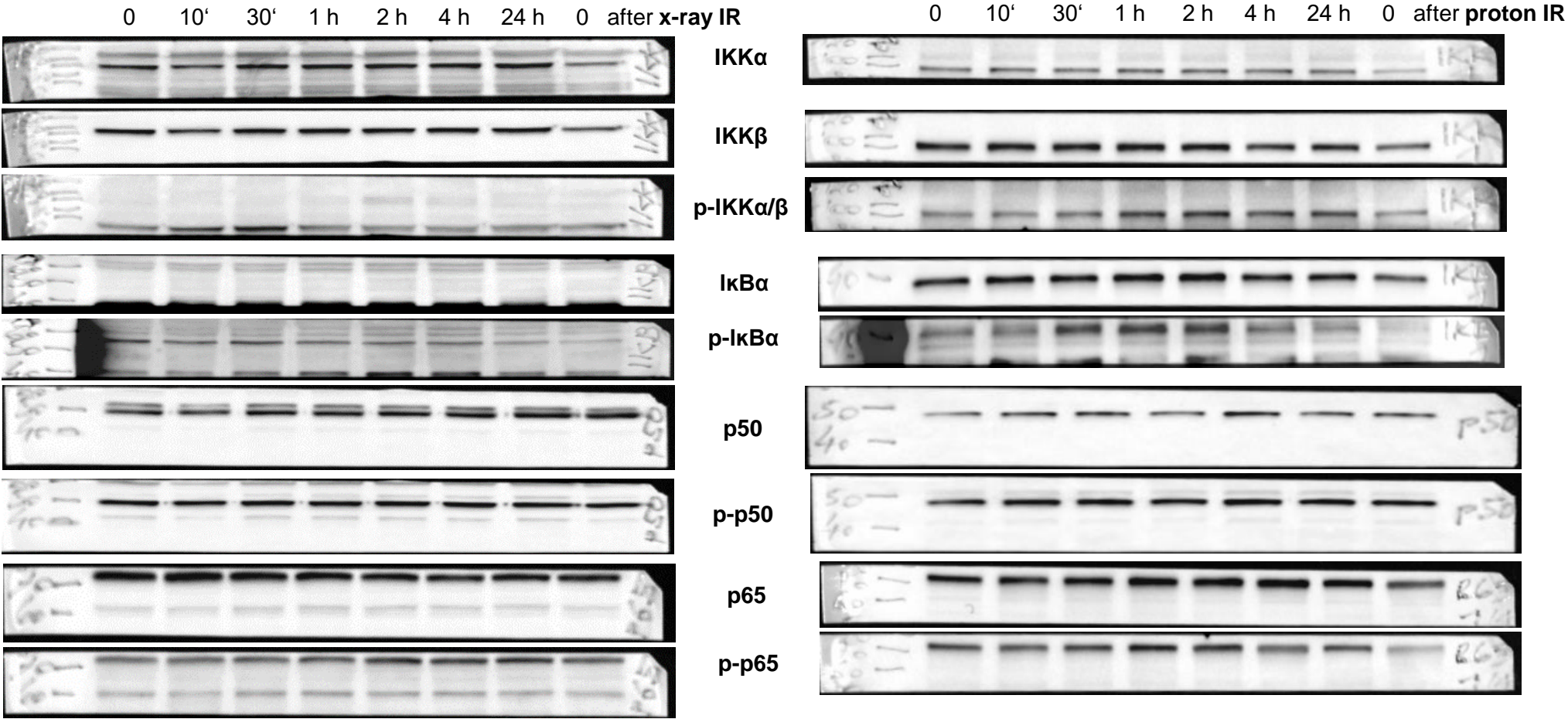

Supplement: Supplementary file 1 — Supplementary Figure S1. [file 41598_2021_3529_MOESM1_ESM.pdf]
